# Sarcopenia in osteoarthritis and rheumatoid arthritis: The association with self-reported fatigue, physical function and obesity

**DOI:** 10.1371/journal.pone.0217462

**Published:** 2019-06-06

**Authors:** Lara Vlietstra, Simon Stebbings, Kim Meredith-Jones, J. Haxby Abbott, Gareth J. Treharne, Debra L. Waters

**Affiliations:** 1 University of Otago, Department of Medicine, Dunedin, New Zealand; 2 University of Otago, Dunedin School of Medicine, Department of Surgical Sciences, Dunedin, New Zealand; 3 University of Otago, Department of Psychology, Dunedin, New Zealand; 4 University of Otago, School of Physiotherapy, Dunedin, New Zealand; University of Tennessee Health Science Center College of Graduate Health Sciences, UNITED STATES

## Abstract

**Aim:**

To determine if there is an association between sarcopenia, physical function and self-reported fatigue in osteoarthritis (OA) and rheumatoid arthritis (RA).

**Methods:**

A cross-sectional analysis of measurements from a cohort of 157 participants with OA or RA was performed. The relationship between muscle mass (appendicular muscle index (AMI)), physical function (timed up and go, 30-seconds sit-to-stand test, 40-meter fast-paced walk test and grip-strength) and two fatigue measures (Multidimensional Assessment of Fatigue (MAF) and a fatigue Visual Analogue Scale (VAS)) was explored using hierarchical linear regression or logistic regression with established AMI cut-offs for sarcopenia.

**Results:**

There were no significant differences for perceived fatigue-related variables between OA or RA sarcopenic or non-sarcopenic participants. Participants with OA had worse physical function (TUG; *P* = 0.029, STS; *P* = 0.004, WS; *P* = 0.003), but participants with RA had lower grip strength (*P*<0.001). The RA group had higher CRP (*P* = 0.006), were more likely to receive glucocorticoids (*P*<0.001), and experienced worse fatigue (*P* = 0.050). The hierarchical multiple regression showed that self-reported fatigue (VAS/MAF-distress) had a significant but weak association with AMI in RA. Participants with higher percentage body fat had a significantly stronger association with sarcopenia in both OA and RA.

**Conclusion:**

Sarcopenia, when assessed by AMI, does not appear to be strongly associated with self-reported fatigue or physical function in participants with either OA or RA. Higher body fat had a moderately strong association with sarcopenia in this cross-sectional study, suggesting that body composition may be an important factor in the health of patients with longstanding OA or RA.

## Introduction

Sarcopenia is defined as the loss of skeletal muscle mass and strength, both of which are subject to a gradual age-related decline [1]. Progressive loss of muscle mass begins as early as 40 years of age and has been estimated at about 8.0% per decade until the age of 70 years [2]. After reaching 70, this loss increases to 15.0% every decade [2] and can eventually result in a 50.0% loss in muscle mass by the age of 80 [3]. A systematic review on the prevalence of sarcopenia by the European Working Group on Sarcopenia in Older People (EWGSOP) reported a prevalence in general community-dwelling older adults of up to 29% [4]. Some major health-related outcomes associated with sarcopenia include osteoporosis [5], obesity [6], dementia [7], type 2 diabetes [8] and lower health-related quality of life [5]. These comorbidities are also detrimental to the overall health of people with osteoarthritis (OA) and rheumatoid arthritis (RA) and result in worse outcomes for patients with these forms of arthritis [9]. Previous research suggests that sarcopenia is common in patients with OA [10] and RA, [11–13] but further research is required to test correlations of sarcopenia in these patients.

Fatigue is a prevalent symptom in patients with OA and RA and qualitative studies suggest this is one of their most troubling symptoms [14]. A 2010 review reported that the prevalence of fatigue in OA varied between 41 and 56%, with 10% experiencing severe fatigue [14]. The prevalence of fatigue in RA ranged from 40 to 80%, with 40% experiencing persistent severe fatigue [14]. In the general population, experiences of fatigue from midlife onwards are associated with declines in physical function and strength [15] and fatigue was also identified as a common reason for patients with OA to avoid physical activity [16]. The association between fatigue and reduced physical activity may lead to loss of strength and function which in turn could speed the progressive loss of muscle mass with an increased risk of developing sarcopenia [17]. Furthermore, individuals with chronic fatigue have been noted to have reduced peak activity levels in part through fear of exacerbating their fatigue which can in turn lead to deconditioning [18].

Infrequent physical activity is common in patients with OA [19] and RA [20] and it is recognised that in OA there is a relationship between avoidance of activity and limitation in activity, which is partly mediated by low muscle strength [21]. However, most research in this area has focused on local muscular atrophy [21] and not specifically on sarcopenia. Assessing generalised age-related loss of muscle mass (i.e. sarcopenia), rather than local muscular atrophy may provide evidence for an association between self-reported fatigue and general loss of muscle mass.

Thus, although sarcopenia and fatigue are independently reported in patients with OA and RA, it remains unclear if there is a relationship between sarcopenia and self-reported fatigue in OA and RA. Such a relationship would be expected from existing literature, since patients with high levels of fatigue have reduced peak activity levels. The present study therefore aimed to investigate the association between sarcopenia and self-reported fatigue in participants with OA and RA while controlling for physical function and other potentially confounding factors. We hypothesized that sarcopenic participants with OA and RA would be less physically active and have higher levels of self-reported fatigue. We also investigated whether measures of physical function, other disease associated variables and self-reported sleep were associated with the presence of sarcopenia.

## Methods

### Design

A cross-sectional data analysis was conducted on a cohort of participants with OA and RA recruited from a public hospital covering a large geographical area in New Zealand.

### Study population

A detailed description of the cohort study has been published previously [22]. We report on the 2010/2011 assessment, comprising 171 participants (n = 87 OA and n = 84 RA), which is 75% of the original cohort [22]. In brief, participants 18 years and older were invited to participate if they fulfilled 1) 1987 The American College of Rheumatology (ACR) criteria for RA or 2) ACR criteria for lower limb OA, defined by pain from either or both knees and/or hips on most days of the previous 3 months, in combination with radiological changes of OA. Any secondary diagnosis that may be associated with fatigue was an exclusion criterion from the original cohort [22]. Participants were excluded from the current study if they had incomplete baseline radiographs (n = 5) or incomplete physical activity measurements (n = 9). Ethical approval for the study was obtained from the Lower South Regional Ethics Committee (LRS/08/30/EXP), Health and Disability Commission, New Zealand. All participants provided written informed consent in accordance with the Declaration of Helsinki.

### Outcome measures

#### Sarcopenia

Sarcopenia was classified by calculating the appendicular muscle index (AMI), defined as the total appendicular lean mass adjusted for body mass index (BMI) (weight/height^2^). The following well-established cut-points were used to define sarcopenia: men with AMI<0.789 and women with AMI<0.512 and were developed and recommended by the Foundation for the National Institutes of Health (FNIH) [23]. In order to not distort the prevalence of sarcopenia, due to the pathologic low grip strength in participants with RA, we applied the FNIH definition.

#### Self-reported fatigue

Two questionnaires were administered to assess self-reported fatigue. The Multidimensional Assessment of Fatigue (MAF) is a 15-item scale [24]. The MAF total score ranges from 1 (no fatigue) to 50 (severe fatigue), with higher scores representing greater fatigue severity, distress, or interference with activities of daily living [24]. The MAF has good internal consistency, construct and criterion validity, reliability and is sensitive to change [24]. Individual sub-scores are also calculated for the four different dimensions of self-reported fatigue measured within the MAF (severity, distress, timing of fatigue, interference with activities of daily living).

A 100mm visual analog scale (VAS) was also used to measure self-reported fatigue. It consists of a 100mm horizontal line, with a question “How would you rate your energy levels?” and two anchoring statements “not tired: full of energy’” and “totally exhausted” [24]. The participants were asked to make a mark or cross on the VAS line that best reflects their level of fatigue over the last seven days. A higher score represents a greater severity or intensity of fatigue [24]. Test-retest reliability has been reported as good in RA, as has construct validity [24].

#### Body composition

Percentage body fat (%), lean mass (kg) and fat mass (kg) of the whole body and arms and legs were assessed using whole-body Dual-energy X-ray Absorptiometry (DXA) (GE Medical Systems, Lunar Prodigy, Madison WI).

#### Physical function

Participants’ mobility was assessed using the timed up and go (TUG) [25] and the lower limb strength by the 30-seconds sit to stand test (STS) [25]. The 40-meter fast-paced walk test (40MWT) was performed to measure walking speed (WS) [25] and grip strength (GS) was assessed using a Jamar Grip Strength Dynamometer in sitting position, whereby participants were given one practice trial and the second trial was recorded (Jamar, Clifton, NJ, USA).

#### Physical activity

Physical activity was measured with a closed-cover pedometer (Braintek) [26]. Participants were asked to wear the pedometer from the time they got dressed until the time they went to bed, for 7 days and recorded their step activity in a diary at the end of each day. The average step count over 7 days was calculated by dividing total steps by the amount of days that the pedometer was worn. Pedometer data was excluded if they reported a step count <3 days of the 7 day period.

#### Disease specific measures of severity/activity

The Western Ontario and McMaster Universities Osteoarthritis Index (WOMAC) [27] was used to measure pain, stiffness and physical functioning in both patient groups. A health assessment questionnaire was used to measure functional disability (Health Assessment Questionnaire—Disability Index, HAQ-DI) [28]. C-reactive protein (CRP-mg/L) [29] was obtained from venous blood samples, to measure whole body systemic inflammatory status in participants in both OA and RA. Disease activity was measured with the Disease Activity Score-28 (DAS-28-CRP) [30], a clinical and laboratory measure of active inflammation, in participants with RA only.

#### Sleep

A self-reported assessment was completed for sleep (VAS sleep) [31] in both OA and RA participants. The question asked was; “How would you rate your average amount of sleep you have had over the last seven days?” with the anchors: “Full night’s sleep” and “No sleep at all”.

### Statistical analysis

Means, standard deviations and confidence intervals were calculated to describe the sample. Outliers were detected using the outlier labelling rule [32]. Correlation coefficients were used to check for multicollinearity and shared variance between the outcomes.

To determine differences in characteristics between OA and RA and to determine group differences between sarcopenic and non-sarcopenic participants with OA and RA, two-tailed two-sample t-tests were used for normally-distributed continuous variables and Mann-Whitney U-tests were used for non-normally distributed continuous variables. Chi-square contingency table analysis was used to determine differences for pairs of categorical variables. The preliminary Levene’s test for equality of variances was used to test the assumption of equal variance.

A hierarchical multiple regression was used to assess the ability of two self-reported fatigue measures (MAF and VAS) to explain continuous levels of AMI, before and after controlling for the influence of age, sex, CRP-levels and steroid usage (model 1). Changes in R^2^ were reported to further determine the contribution of the additional variables of self-reported fatigue. Also, a logistic regression was performed to test the association between sarcopenia and sex, percentage body fat, disease specific measures of severity/activity, use of glucocorticoid steroids, measurements of self-reported fatigue, health assessment and functional disability, physical activity and physical function, all one by one, due to the low number of sarcopenic participants.

Data analysis was performed using the Statistical Package for the Social Sciences (SPSS 22.0 for Windows, SPSS Inc., Chicago, USA).

## Results

### Descriptive statistics

The demographic characteristics of the two disease cohorts are reported in Table 1. Participants with OA were significantly older (*P* = 0.001), had higher BMI (*P*<0.001) and worse physical function results (TUG; *P* = 0.029, STS; *P* = 0.004, WS; *P* = 0.003), whereas participants with RA had higher CRP-scores (*P* = 0.006), were more likely to be prescribed glucocorticoid-steroids (*P*<0.001), experienced worse severity of fatigue (*P* = 0.050) and had lower grip strength (*P*<0.001). Sarcopenia was present in 29.3% of the patients with OA and in 17.1% of the patients with RA (*P* = 0.071). Physical activity levels were not significantly different between patients with OA and RA (P = 0.489).

**Table 1:** Participant characteristics.

	OA (n = 75)		RA (n = 82)		
	Mean (SD)	CI	Mean (SD)	CI	p-value
Age (years)	**68.8 (8.9)**	**66.8–70.9**	**61.1 (13.3)**	**58.2–64.0**	<0.001*
Sex (%female)	60 (-)		73.2 (-)		0.082
BMI	**30.8 (6.3)**	**29.3–32.2**	**27.5 (5.1)**	**26.4–28.6**	<0.001*
Body fat (%)	38.8 (8.9)	36.7–40.8	36.9 (10.0)	34.7–39.1	0.214
AMI (kg)	0.7 (0.2)	0.65–0.74	0.7 (0.2)	0.6–0.7	0.950
CRP (mg/l)	**4.6 (2.3)**	**4.06–5.11**	**8.8 (13.3)**	**5.9–11.7**	0.006*
Sarcopenia (%sarcopenic)	29.3 (-)		17.1 (-)		0.071
Steroids (%using)	**0 (-)**		**42.7 (-)**		<0.001*
Fatique (MAF, points)	21.1 (10.3)	18.7–23.4	24.1 (10.9)	21.7–26.4	0.064
MAF, severity	**9.0 (4.6)**	**7.9–10.1**	**10.5 (4.9)**	**9.4–11.6**	0.050*
MAF, distress	3.0 (2.5)	2.5–3.6	3.6 (2.8)	3.0–4.2	0.165
MAF, DL	3.0 (1.8)	2.6–3.4	3.3 (2.0)	2.9–3.7	0.239
MAF, timing	11.4 (4.1)	10.5–12.4	12.5 (3.5)	11.8–13.	0.057
Fatigue (VAS, mm)	39.2 (24.4)	33.6–44.8	43.3 (23.8)	38.1–48.6	0.283
WOMAC (points)	30.5 (19.1)	26.1–34.9	26.3 (18.6)	22.2–30.4	0.161
DAS28 (points)			3.6 (1.3)	3.3–3.8	
HAQ-DI (points)	0.9 (0.9)	0.8–1.1	1.0 (0.7)	0.9–1.1	0.534
Pedometer (steps/day)	4375 (2587)	3749–5001	4700 (2913)	4010–5389	0.489
TUG (sec)	**7.8 (2.9)**	**7.2–8.5**	**6.8 (2.8)**	**6.2–7.5**	0.029*
STS (number)	**10.0 (3.8)**	**9.1–10.1**	**11.8 (3.8)**	**11.0–12.7**	0.004*
Walking speed (m/s)	**1.3 (0.3)**	**1.2–1.4**	**1.5 (0.3)**	**1.4–1.5**	0.003*
Grip Strength (kg)	**26.0 (11.3)**	**23.4–28.7**	**18.2 (8.9)**	**16.2–20.2**	<0.001*

OA = osteoarthritis, RA = Rheumatoid Arthritis, n = number, SD = Standard Deviation, CI = Confidence Interval, BMI = Body Mass Index, AMI = Appendicular Muscle Index, CRP = C-reactive protein, mg/l = milligram per litre, MAF = Multidimensional Assessment of Fatigue, DL = Daily Living, VAS = Visual Analog Scale, mm = millimetres, WOMAC = Western Ontario and McMaster Universities Osteoarthritis Index, DAS-28 = Disease Activity Score 28, HAQ-DI = Health Assessment Questionnaire Disability Index, TUG = Timed Up and Go, Sec = seconds, STS = Sit To Stand, m/s = meter per second, kg = kilograms. Data are mean (SD) unless otherwise stated.

* significant difference between groups = p < .05

### Differences between sarcopenic and non-sarcopenic participants

There were no significant differences between sarcopenic and non-sarcopenic participants with OA or RA across the range of perceived fatigue related and physical activity related variables (Table 2). However, both BMI and percentage body fat were significantly higher in OA sarcopenic participants compared with non-sarcopenic participants with OA (*P*<0.001 and *P*<0.001 respectively) and this was also the case in participants with RA (*P*<0.001 and *P* = 0.004 respectively). CRP-levels were higher in sarcopenic participants with OA compared to non-sarcopenic participants with OA (*P* = 0.048). Sarcopenic participants with OA also scored slower on the TUG (*P* = 0.036), had slower gait speed (*P* = 0.005) and had lower grip strength (*P* = 0.029) compared with non-sarcopenic participants with OA. Sarcopenic participants with RA were significantly older (p = 0.08), had higher CRP-levels (*P* = 0.003) and had significantly lower gait speed (P = 0.041) compared to non-sarcopenic patients.

**Table 2:** Comparison between variables in relation to the presence of sarcopenia in OA and RA.

	OA (n = 75)			RA (n = 82)		
	Sarcopenic (n = 22)	Non- sarcopenic	p-value	Sarcopenic (n = 14)	Non- sarcopenic	p-value
Age	71.6	67.7	0.087^	**69.4**	**59.4**	0.008*^
Sex (%female)	64% (n = 14)	58% (n = 31)	0.798	64% (n = 9)	75% (n = 51)	0.509
BMI	**34.4**	**29.3**	<0.001^*	**32.5**	**26.5**	<0.001*^
Body fat (%)	**44.7**	**36.3**	<0.001^*	**43.4**	**35.5**	0.004*^
CRP-levels	**5.1**	**4.4**	0.048^*	**14.3**	**7.7**	0.003^*
Steroids				64% (n = 9)	38% (n = 26)	0.073
Fatigue (MAF)	21.4	20.9	0.869	24.2	24.2	0.983
MAF, severity	8.8	9.1	0.820^	10.1	10.6	0.786^
MAF, distress	3.2	3.0	0.847^	4.4	3.5	0.373^
MAF, DL	3.2	2.9	0.629^	3.1	3.4	0.786^
MAF, timing	11.9	11.2	0.268^	12.0	12.7	0.747^
Fatigue (VAS)	39.1	39.2	0.848^	44.5	43.1	0.848^
WOMAC	32.0	29.9	0.727^	29.8	25.6	0.286^
HAQ-DI	1.1	0.8	0.081^	1.1	1.0	0.464^
Pedometer	3522	4730	0.065^	3921	4843	0.382^
Physical Function						
TUG (sec)	**8.7**	**7.4**	0.036^*	8.2	6.6	0.090^
STS (number)	10.1	10.0	0.703^	10.00	12.2	0.054^
Walking speed (m/s)	**1.2**	**1.4**	0.005*	**1.3**	**1.5**	0.041*
Grip Strength (kg)	**21.7**	**27.9**	0.014^*	14.9	18.8	0.148^

OA = osteoarthritis, RA = Rheumatoid Arthritis, n = number, BMI = Body Mass Index, CRP = C-reactive protein, MAF = Multidimensional Assessment of Fatigue, DL = Daily Living, VAS = Visual Analog Scale, WOMAC = Western Ontario and McMaster Universities Osteoarthritis Index, HAQ-DI = Health Assessment Questionnaire Disability Index, TUG = Timed Up and Go, STS = Sit To Stand, WS = Walking Speed, GS = Grip Strength. Data are mean (SD) unless otherwise stated.

* significant difference between sarcopenic and non-sarcopenic = p < .05.

^ Mann-Whitney U- test

### Appendicular muscle index

The results of the hierarchical regression analysis are shown in Table 3. The self-reported total fatigue score on the MAF explained 3.6% of the variance of AMI in OA and 3.5% in RA (*P* = 0.101 and *P* = 0.090, respectively). Self-reported fatigue on the VAS explained 2.2% (*P* = 0.201) of the variance of AMI in OA but significantly more in RA 7.4% (*P* = 0.013). Unadjusted univariate analysis of the MAF subscales showed that only distress resulted in a significant explanation of AMI in RA, explaining 5.3% of the variance (*P* = 0.038). However, after adjusting for age, sex, CRP-levels and steroids use, self-reported fatigue (MAF) did not explain any of the variance of AMI in OA and only 0.4% in RA. Using a VAS to measure fatigue perception did not change these results, with 2.2% of the variance explained in OA and only 7.4% in RA in the unadjusted model. After adjusting for age, sex, CRP-levels and glucocorticoid-steroid use, the perceived fatigue-related distress subscale of the MAF explained a significant 1.8% of the variance in AMI in RA (p = 0.045).

**Table 3:** Summary of the unadjusted and adjusted hierarchical multiple regression for appendicular muscle index and fatigue.

	Unadjusted		Adjusted^a^			
	R^2^	Sig.	R^2^	Sig.	R^2^ Change	Sig.
OA						
Fatigue (MAF)	0.036	0.101	0.582	<0.001*	0.000	0.794
Fatigue (MAF, severity)	0.031	0.133	0.582	<0.001*	0.000	0.924
Fatigue (MAF, distress)	0.026	0.168	0.582	<0.001*	0.000	0.837
Fatigue (MAF, DLA)	0.031	0.131	0.582	<0.001*	0.000	0.779
Fatigue (MAF, timing)	0.017	0.262	0.583	<0.001*	0.001	0.683
Fatigue (VAS)	0.022	0.201	0.582	<0.001*	0.000	0.944
RA						
Fatigue (MAF)	0.035	0.090	0.664	<0.001*	0.004	0.348
Fatigue (MAF, severity)	0.022	0.180	0.662	<0.001*	0.001	0.575
Fatigue (MAF, distress)	**0.053**	0.038*	**0.678**	<0.001*	**0.018**	0.045*
Fatigue (MAF, DLA)	0.041	0.067	0.663	<0.001*	0.002	0.458
Fatigue (MAF, timing)	0.009	0.388	0.662	<0.001*	0.001	0.640
Fatigue (VAS)	**0.074**	0.013*	0.671	<0.001*	0.011	0.120

CRP = C-reactive protein, OA = osteoarthritis, RA = Rheumatoid Arthritis, Sig. = Significant, bisuse = bisphosphonate use, MAF = Multidimensional Assessment of Fatigue, DLA = Daily Living Activities, VAS = Visual Analog Scale.

^a^ = adjusted for age, sex, CRP-levels and steroid use in model 1

* significant = p < .05

### Associations with sarcopenia

The adjusted odds ratios (ORs) for sarcopenia in the OA and RA groups are shown in Table 4. Within the participants with OA, those who had higher percentage body fat or low gait speed were more likely to be sarcopenic (OR: 1.2, 95%CI: 1.09–1.32, P<0.001 and OR: 0.13; CI: 0.02–0.83; *P* = 0.03, respectively). Participants with RA who had higher percentage body fat had 1.1 higher odds (95%CI: 1.0–1.2) of being sarcopenic compared to those with lower percentage body fat (*P* = 0.02) and participants who were using steroids were 1.08 times (95%CI: 1.0–1.2) more likely of being sarcopenic (*P* = 0.017) compared to those not using glucocorticoid steroids.

**Table 4:** Summary of logistic regression analysis for sarcopenia, adjusted for age.

	OA (n = 75)			RA (n = 82)		
	OR	CI	p-value	OR	CI	p-value
Sex (female)	0.9	0.3–2.6	0.851	1.7	0.5–6.2	0.420
Body fat (%)	**1.2**	**1.1–1.3**	<0.001*	**1.1**	**1.0–1.2**	0.019*
C-reactive protein (mg/l)	1.1	0.9–1.4	0.338	1.0	1.0–1.1	0.102
Steroids (yes)	1.1	1.0–1.1	0.090	**1.1**	**1.0–1.2**	0.017*
Fatigue (MAF) (point)	1.0	1.0–1.1	0.867	1.0	1.0–1.1	0.878
MAF, severity	1.0	0.9–1.1	0.773	1.0	0.9–1.1	0.640
MAF, distress	1.0	0.9–1.3	0.713	1.2	1.0–1.5	0.106
MAF, DL	1.1	0.8–1.4	0.606	0.9	0.7–1.3	0.677
MAF, timing	1.1	0.9–1.2	0.369	1.0	0.8–1.7	0.760
Fatigue (VAS) (mm)	1.0	1.0–1.0	0.737	1.0	1.0–1.0	0.664
WOMAC (point)	1.0	1.0–1.0	0.544	1.0	1.0–1.0	0.716
DAS-28 (point)				1.5	0.9–2.5	0.101
HAQ-DI (point)	1.8	0.8–4.0	0.132	1.0	0.4–2.4	0.933
Pedometer (step)	1.0	1.0–1.0	0.163	1.0	1.0–1.0	0.721
TUG (sec)	1.1	0.9–1.4	0.186	1.1	0.9–1.3	0.594
STS (number)	1.0	0.9–1.2	0.900	0.9	0.7–1.1	0.286
Walking speed (m/s)	**0.1**	**0.0–0.8**	0.030*	0.3	0.0–3.7	0.366
Grip Strength (kg)	1.0	0.9–1.0	0.106	1.0	0.9–1.1	0.493

OA = osteoarthritis, RA = Rheumatoid Arthritis, n = number, OR = Odds Ratio, CI = Confidence Interval, mg/l = milligram per litre, MAF = Multidimensional Assessment of Fatigue, DL = Daily Living, VAS = Visual Analog Scale, WOMAC = Western Ontario and McMaster Universities Osteoarthritis Index, DAS-28 = Disease Activity Score 28, HAQ-DI = Health Assessment Questionnaire Disability Index, TUG = Timed Up and Go, STS = Sit To Stand, sec = second, m/s = meter per second, kg = kilogram.

* significant = p < .05

## Discussion

The results of this research suggest that sarcopenia is not strongly associated with self-reported fatigue in either OA or RA. However, significant associations between physical function and inflammatory measures and sarcopenia were apparent in both OA and RA participants. Furthermore, higher percentage body fat was significantly association with sarcopenia in both OA and RA participants. These findings have added to the limited knowledge about correlations of sarcopenia in these populations and have potential clinical implications for the screening and provision of care in rheumatology.

The differences in participant characteristic between the OA and RA groups were as expected. RA patients were slightly younger, which would be expected as RA manifests itself at a younger age [33], whereas OA is an age-related process and therefore manifests itself at a later stage of life [34]. Furthermore, participants with RA had significantly higher CRP levels and greater use of medications, much of which can be explained by the inflammatory nature of the RA disease process as standard treatment involves the use of anti-inflammatory and immunosuppressant therapies [35]. Finally, the lower BMI seen in the participants with RA was expected since the inflammatory process of RA is driven by pro-inflammatory cytokines which alter energy metabolism [36].

High percentage body fat was significantly associated with sarcopenia in both OA and RA. These results are supported by earlier research that reported a positive correlation between obesity and sarcopenia and can be explained by the fact that when fat mass increases, there is a smaller lean muscle to fat ratio, often referred to as sarcopenic-obesity (SO) [37]. In the present sample 72% of the participants with OA (n = 54) and 57.3% of the participants with RA (n = 47) were obese, based on percentage body fat (Men>30%, Women>40%) [38]. Using the AMI cut-off scores for sarcopenia and the fat percentage cut-off scores for obesity, 75.9% of the participants with OA and sarcopenia (n = 22) and 76.5% of the participants with RA and sarcopenia (n = 13) were obese and have therefore SO. These results are similar to Lemmey et al., who reported that patients with RA had higher weight, higher BMI, greater mean waist circumference and 10% less muscle than controls without RA [12]. Research by Morley et al. has shown that persons who are both obese and sarcopenic have worse functional impairment, disability, and more falls independent of age, than those who are sarcopenic and non-obese [39]. This high percentage of sarcopenia, obesity and SO in both OA and RA identifies a significant comorbidity which is under recognised but may have profound effects on mobility and general health. Raising awareness and screening patients with OA and RA for sarcopenic obesity should be considered and also underlines the urgent need for further research.

Another significant association of sarcopenia in RA in this study was current treatment with corticosteroids. Glucocorticoids are acknowledged to have a potent effect on muscle metabolism leading to reduced muscle mass, which can be a serious side-effect of treatment with corticosteroids [40]. However, these results are contradictory to earlier published research on sarcopenia and RA, which did not report a relationship between corticosteroid use and sarcopenia [13, 41].

Physical function measures such as TUG, walking speed, and grip strength in the OA group and walking speed in the RA group, were significantly worse in participants with sarcopenia. Although these measures of physical function were not statistically different between sarcopenic and non-sarcopenic patients, they did reach levels of clinically significant difference. We have therefore reported minimal clinically important differences (MCIDs). Sarcopenic OA participants had clinically significant lower scores on the TUG (MCID = 1.2 seconds [42]) and walking speed (MCID = 0.2 meter/second [42]). Within RA patients, there were clinically significant differences between sarcopenic and non-sarcopenic participants for TUG (MCID = 1.2 seconds [42]), STS (MCID = 2.3 repetitions [42]) and walking speed (MCID = 0.2 meter/second [42]). These clinically meaningful differences may indicate a trend towards a true relationship and highlight the importance within the clinical setting. The wide confidence intervals, and lack of statistical significance are potentially attributable to low power and further studies with larger numbers of participants are warranted to further explore these associations.

Hierarchical regression analysis comparing self-reported fatigue outcomes and AMI showed that the VAS and the distress subscale of the MAF in RA had a significant, but small association with AMI. The other hierarchical regression analysis did not demonstrate significant associations in OA, but did show a trend in the expected direction. Although both self-reported fatigue measurement instruments are known to be reliable [24], the VAS is an one-dimensional concept and hence perhaps more sensitive in the context of this study [43].

Significant differences in CRP levels between sarcopenic and non-sarcopenic participants were expected, as higher CRP levels in sarcopenic participants in both patients with OA and RA has been demonstrated by others [44]. A recent systematic review also suggests that inflammatory cytokines prompt muscle wasting, ultimately stimulating protein catabolism and suppressing muscle synthesis [45]. The significant lower physical function measurements were also expected, perhaps as a result of functional deficits [46] but a significant association between sarcopenia and functional disability was not evident in the present cross-sectional study.

Previous research has suggested a relationship between physical activity and sarcopenia [47]. However, like the present study, previous studies are limited by their cross-sectional design and therefore longitudinal research is now needed to further explore these relationships. The results of the current study are in line with the previous published studies and, although not statistically significant, suggest that sarcopenic participants with OA and RA tend to be less physically active (as measured by seven-day pedometer recordings) compared to non-sarcopenic participants. Lower physical activity level could hasten the progression of sarcopenia and may confound the relationship between self-reported fatigue and sarcopenia for patients with OA and RA. Future longitudinal research with larger sample sizes should aim to elucidate the mechanisms that underlie the relationship between sarcopenia, self-reported fatigue and physical activity.

The prevalence of sarcopenia in RA in the current study was lower than previously reported. Doğan et al. [41] reported a prevalence of sarcopenia of 43.3% while Giles et al. [13] reported a prevalence of 25.9%. Using different definitions of sarcopenia could have influenced these outcomes. The classification of sarcopenia in this current research was based on AMI/BMI (Males <0.789, Females <0.512) criteria as proposed by the FNIH [23], whereas Doğan et al. and Giles et al. based their definition on relative skeletal muscle index cut-scores (Males ≤8.50 kg/m^2^, Females ≤5.75 kg/m^2^), which is not specific to AMI [13, 41]. We applied the AMI/BMI definition instead of other definitions that include grip strength [48], because grip strength in our participants with RA was very low, which could have distorted the prevalence of sarcopenia. This is illustrated by our results, showing that participants with RA had significantly lower grip strength when compared to participants with OA, despite having similar levels of ALM, which could have been caused by localised pain in the hands. Also, a recently published paper on the same cohort showed that hand function is more important than pain in affecting grip [49]. Although this could be viewed as a possible limitation of this study, there is still little agreement on sarcopenia cut-scores and even less is known about the most appropriate sarcopenia definitions in disease specific populations.

The main limitation of this study was the low number of sarcopenic participants within the sample. However, we attempted to control for this in the hierarchical multiple regression, by avoiding over specification of the models [50]. Despite the small sample size, non-significant but clinically meaningful differences were apparent, which supports the need for larger studies with greater power to further explore these differences. Also, because we were interested in the multivariate predictors of sarcopenia, we treated sarcopenia as the outcome and acknowledge that a reverse relationship cannot be excluded. Furthermore, due to the cross-sectional nature of this study, relationships between self-reported fatigue, sarcopenia and physical activity levels could not be explored for causal associations. In order to develop interventions to mitigate the fatigue experienced by participants with OA and RA, longitudinal research is needed to explore the manner in which these variables interact.

Despite these limitations, there are several strengths of our study. Fatigue is an inherently difficult variable to measure and therefore we included the MAF, which was developed to explore four different aspects of fatigue (degree, distress, impact and timing) and concurrently measured self-reported fatigue using a VAS, which is as valid and reliable as more complex measures of fatigue, yet it is easier to use [43]. We also included both inflammatory and non-inflammatory types of arthritis, as well as combining subjective fatigue data with objectively measured physical activity data.

## Conclusion

In conclusion, sarcopenia, assessed by AMI, does not appear to be strongly associated with self-reported fatigue based on the findings of this cross-sectional study. However, a high percentage body fat was significantly associated with sarcopenia in both types of arthritis, which is an important association that deserves further exploration. Future longitudinal research with larger purposive subsamples of OA and RA patients with sarcopenia, is needed to confirm these findings. In clinical practice the possibility of sarcopenia should be considered in patients who are obese, who are treated with glucocorticoids, or who have unsuppressed inflammation.
